# Diagnostic Efficacy of Cell Block and Liquid-Based Cytology for Endoscopic Ultrasound-Guided Fine Needle Aspiration in Pancreatic Tumors

**DOI:** 10.5152/tjg.2024.23609

**Published:** 2024-08-01

**Authors:** Ali Şenkaya, Ferit Çelik, Fatih Tekin, İlker Turan, Deniz Nart, Nevin Oruç, Ahmet Aydın

**Affiliations:** 1 Department of Gastroenterology, University of Health Sciences İzmir Bozyaka Training and Education Hospital, İzmir, Türkiye; 2 Department of Gastroenterology, Bakırçay University Çiğli Training and Education Hospital, İzmir, Türkiye; 3 Division of Gastroenterology, Department of Internal Medicine, Ege University, Faculty of Medicine, İzmir, Türkiye; 4 Department of Pathology, Ege University, Faculty of Medicine, İzmir, Türkiye

**Keywords:** Endoscopic ultrasound-guided fine needle aspiration, pancreatic neoplasms, cytopathology, diagnosis

## Abstract

**Background/Aims:**

This study aimed to evaluate the diagnostic efficacy of cell block (CB) and liquid-based cytology (LBC) for endoscopic ultrasound-guided fine-needle aspiration (EUS-FNA) in pancreatic tumors.

**Materials and Methods:**

The study included patients who underwent EUS-FNA for pancreatic tumors between January 2015 and February 2021 and whose cytology samples were both processed for LBC and CB.

**Results:**

Data of 390 patients (220 men, mean age: 64.2 ± 11.4 years) were retrospectively analyzed. Of the detected lesions (size: 17-120 mm; mean: 39.9 ± 13.9 mm), 220 (56.4%) were located in the head and uncinate process of the pancreas. Lesions in 339 (86.9%) patients were diagnosed as malignant using CB and/or LBC and suspicious for malignancy in 44 (11.3%) patients. In 7 patients with non-diagnostic (6 cases) or negative for malignancy (1 case) EUS-FNA results using both methods, the diagnosis of malignancy was established via ultrasound-guided percutaneous biopsy. Malignancy was detected in 324 (92.4%), 313 (87.9%), and 298 (87.9%) patients using CB, LBC, and both CB and LBC, respectively. Final diagnosis was obtained in 339 (98%) patients by using CB and/or LBC. The combined use of the both methods exhibited significantly superior diagnostic accuracy compared with CB and LBC alone (*P* < .001).

**Conclusion:**

Liquid-based cytology and CB exhibit high diagnostic accuracy for the detection of pancreatic tumors in patients undergoing EUS-FNA. The combined use of both methods showed a significantly higher diagnostic accuracy than LBC and CB alone.

**Main Points**

- Smear cytology (SC), liquid-based cytology (LBC), and cell block (CB) preparation are commonly used techniques to analyze specimens obtained using endoscopic ultrasound-guided fine-needle aspiration (EUS-FNA).
- LBC and CB exhibit high diagnostic accuracy for the detection of pancreatic tumors in patients undergoing EUS-FNA.
- The combined use of both methods showed a significantly higher diagnostic accuracy than LBC and CB alone.
- The use of LBC in combination with CB may be considered a favorable choice to increase the diagnostic accuracy and reduce loss of time.

## Introduction

Endoscopic ultrasound-guided fine needle aspiration (EUS-FNA) is widely used in the cytopathological diagnosis of pancreatic tumors.^1,2^ Recently, EUS-FNA was reported to have a sensitivity, specificity, and diagnostic accuracy of 85%-98%, 95%-100%, and 91%-98%, respectively, for pancreatic solid tumors.^3,4^ The diagnostic performance of EUS-FNA cytology varies depending on the experience of the clinician performing endoscopy and cytopathology, availability of a cytopathology expert for rapid on-site evaluation (ROSE), lesion characteristics, and evaluation method.^3,5^ Although performing ROSE significantly increases the diagnostic value, it is not feasible in many centers.^6^ Needles of different shapes have been developed for fine needle biopsy (FNB) to obtain samples showing tissue structure^7^ and overcome inadequacies in tissue diagnosis. Some studies showed significantly better diagnostic performance of FNB over FNA in pancreatic lesions; however,^8-12^ the application of FNB needles is limited by their unavailability in every center.

Smear cytology (SC), liquid-based cytology (LBC), and cell block (CB) preparation are commonly used techniques to analyze specimens obtained using EUS-FNA. Liquid-based cytology allows automated slide preparation based on filtration with a uniform, monolayer distribution of cells. Liquid-based fixatives remove red blood cells, mucus, and protein deposits in the background.^13^ Reportedly, CB has a greater contribution to the diagnosis than any method alone, and the application of immunocytochemistry is remarkably useful.^14,15^

In our clinic, both CB and LBC are routinely used for testing pancreatic tumor samples collected via EUS-FNA. This study aimed to compare both methods and evaluate their contribution in the diagnosis of pancreatic malignancies.

## Materials and Methods

Patients who met the following criteria were included in this study: patients aged ≥18 years who underwent EUS-FNA for the diagnosis of pancreatic malignancy in the Endoscopy Unit of the Gastroenterology Department of Ege University Medical Faculty Hospital between January 2015 and February 2021 and whose cytology specimens were both fixed in a special solution for LBC and in alcohol–formalin solution for CB preparation and sent to the pathology laboratory for analyses. Patients who underwent EUS-FNA for purely cystic lesions and those with insufficient data were excluded from the study. The patients’ age, sex, lesion size and location in the pancreas, and pathology results (both LBC and CB) were recorded by searching the hospital database.

A linear echoendoscope (EG 530 UT Fujifilm, Japan or UCT180, Olympus, Center Valley, Pa, USA with Hitachi Aloka Alpha 7 system, Tokyo, Japan) was used for the procedure. Informed consent was obtained from the patients, and the procedure was initiated by sedating the patients after 12 hours of fasting. The coagulation parameters were checked before performing the procedure. Standard 22-gauge needles (Boston Scientific, Natick, Mass, USA or Wilson-Cook, Winston-Salem, NC, USA) were used for EUS-FNA. In each case, material was taken from the existing lesions by performing 2-4 passes (mean, 3) during EUS-FNA. The material was transferred to a tube containing ThinPrep fixation solution [CytoLyt (Cytyc Corp - Boxborough, Mass, USA)] for LBC and alcohol–formalin solution for CB and sent to the pathology laboratory. For LBC, the material in CytoLyt solution was prepared using a ThinPrep5000 automated slide processor (Hologic, Marlborough, Mass, USA). All slides were stained with Papanicolaou stain. The cytologic material fixed in alcohol–formalin was first directly centrifuged. Approximately, 10 mL of material was pipetted from the bottom of the cytologic material into a Falcon tube, and the entire sample was collected if the volume was <10 mL. After adding 25-30 mL of 96% alcohol and 10% formaldehyde in equal proportions, centrifugation was performed at 1660 rpm for 10 minutes. After centrifugation, the supernatant was discarded. The alcohol–formalin mixture was subsequently added again, and the tube was left for a few hours to allow precipitate formation. The precipitate was collected in a tissue cassette and subjected to tissue analysis. Sections of 4 microns were taken from the obtained paraffin blocks and stained with hematoxylin and eosin, and histopathological examination was performed. Cytopathological examinations were performed by an experienced cytopathologist, more than 10 years, blindly.

The pathology results of the patients were classified according to the classification of the Papanicolaou Society of Cytopathology System for Reporting Pancreaticobiliary Cytology (PSCPC). According to this system, category I is non-diagnostic, II is negative (for malignancy), III is atypical, IV is neoplastic: benign or other, V is suspicious for malignancy, and VI is positive/malignant.^16^

Approval was obtained from the Ege University Ethics Committee (approval number: 21-5T/107, date: May 20, 2021) before starting the study. This study was conducted in accordance with the principles of the Declaration of Helsinki on Human Rights.

**Statistical Analysis**

The Statistical Package for the Social Sciences 25.0 program (IBM Corp., Armonk, NY, USA) was used for the analysis of variables. Conformity of data to normal distribution was evaluated using Shapiro–Wilk and Shapiro–Francia tests, and homogeneity of variance was evaluated using Levene’s test. To compare the 2 independent groups according to quantitative data, the Mann–Whitney *U*-test was used in combination with the Monte Carlo method. The categorical variables were compared using Pearson’s chi-square, Fisher Exact, and Fisher–Freeman–Halton tests along with the Monte Carlo simulation technique. Furthermore, the column ratios were compared with each other and analyzed using the Benjamini–Hochberg method with adjusted *P*-value results. Quantitative variables were expressed as mean (± standard deviation), median (minimum/maximum), and median [percentile 25 (q1)/ percentile 75 (q3)], whereas categorical variables were expressed as n (%). Variables were analyzed at a 95% confidence level, and *P* <.05 indicated statistical significance.

## Results

Overall, 14 patients were excluded because they underwent EUS-FNA for the analysis of suspected mass based on their diagnosis of chronic pancreatitis and no malignancy was detected; furthermore, 10 patients were excluded because of insufficient data. The results of these 14 patients were discussed in a multidisciplinary hepatobiliary council and a consensus was reached that malignancy was absent and no findings indicating malignancy were detected in at least 12 months of follow-up. After excluding these cases, the data of 390 patients (220 [56.4%] male; 170 [43.5%] female; mean age, 64.2 [22–87] years) who met the inclusion criteria were retrospectively analyzed. The demographic and clinical characteristics of the patients and the CB/LBC results are shown in Table 1.

**Figure 1.**
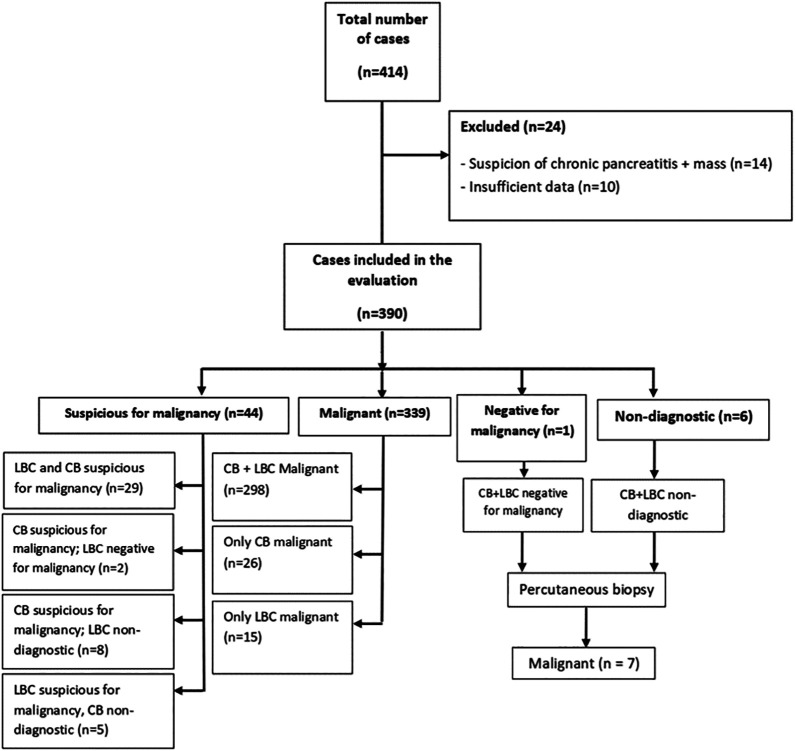
Flowchart of the study.

Of the 390 patients who underwent EUS-FNA, the lesions in 339 (86.9%) were cytologically diagnosed as malignant. Notably, lesions in 298 (87.9%) patients were diagnosed as malignant using both CB and LBC. Furthermore, lesions were diagnosed as malignant in 26 (7.7%) patients using CB alone and in 15 (4.4%) using LBC alone. Lesions diagnosed as malignant via CB alone were found to have different results when diagnosed using LBC alone: 2 were negative for malignancy, 14 were non-diagnostic, and 10 were suspicious for malignancy. However, lesions in 15 patients diagnosed as malignant via LBC were diagnosed as non-diagnostic using CB. Notably, the lesions in 44 (11.3%) patients could not be diagnosed as malignant by either CB or LBC. Suspicious for malignancy was diagnosed in 29 patients using both CB and LBC. Additionally, lesions in 2 patients were diagnosed as suspicious for malignancy using CB and negative for malignancy using LBC. Furthermore, lesions in 8 patients were diagnosed as suspicious for malignancy using CB and non-diagnostic using LBC, and those in 5 patients were diagnosed as suspicious for malignancy using LBC and non-diagnostic using CB. Suspicious for malignancy detected using CB and/or LBC was diagnosed in inoperable patients with locally advanced stage and/or distant metastasis. These cases were evaluated by a multidisciplinary hepatobiliary council using clinical, laboratory, and imaging findings and were considered malignant. Consequently, these patients were referred to the oncology department for treatment. Seven patients were diagnosed as malignant via ultrasound-guided percutaneous biopsy after EUS-FNA results were inconclusive in 6 cases and benign in one case using both methods (Figure 1).

To reveal the possible effects of suspicious for malignancy on the comparison results, statistical analyses were performed by considering cases with suspicion for malignancy as malignant and subsequently excluding those cases. In both the aforementioned scenarios, the use of both CB and LBC significantly enhanced the diagnostic accuracy compared with the use of CB and LBC alone (*P* < .001). When patients with suspicious for malignancy were considered malignant, the diagnostic accuracy of CB was slightly higher than that of LBC (*P* = .045) (Table 2).

In the statistical evaluation of the diagnostic accuracies obtained using both methods in relation to the patients’ age, sex, size, pancreatic location, and sonographic appearance of the lesion, no significant difference was observed in parameters other than age (Table 3). Statistical evaluation of CB and LBC separately in terms of age, sex, lesion size and location in the pancreas, and sonographic appearance revealed a significant correlation between LBC diagnostic accuracy and age and sonographic appearance of the lesion. The diagnostic accuracy of LBC was significantly higher for solid tumors than for solid-cystic tumors (*P* = .031) (Table 4).

**Figure 2.**
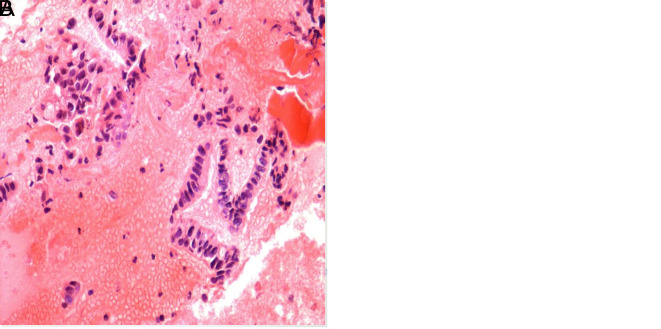
(A) Adenocarcinoma. In the LBC material, tumor cells compatible with adenocarcinoma with an increased nucleus–cytoplasm ratio, nuclear hyperchromasia, and prominent nucleoli, adjacent to the normal duct epithelial plate (Papanicolaou stain; 20× magnification), (B) Cell block material of the same case. Adenocarcinoma cells with hyperchromatic, nuclear membrane irregularity in the blood and fibrinoid background (hematoxylin–eosin stain; 40× magnification). LBC, liquid-based cytology.

**Figure 3.**
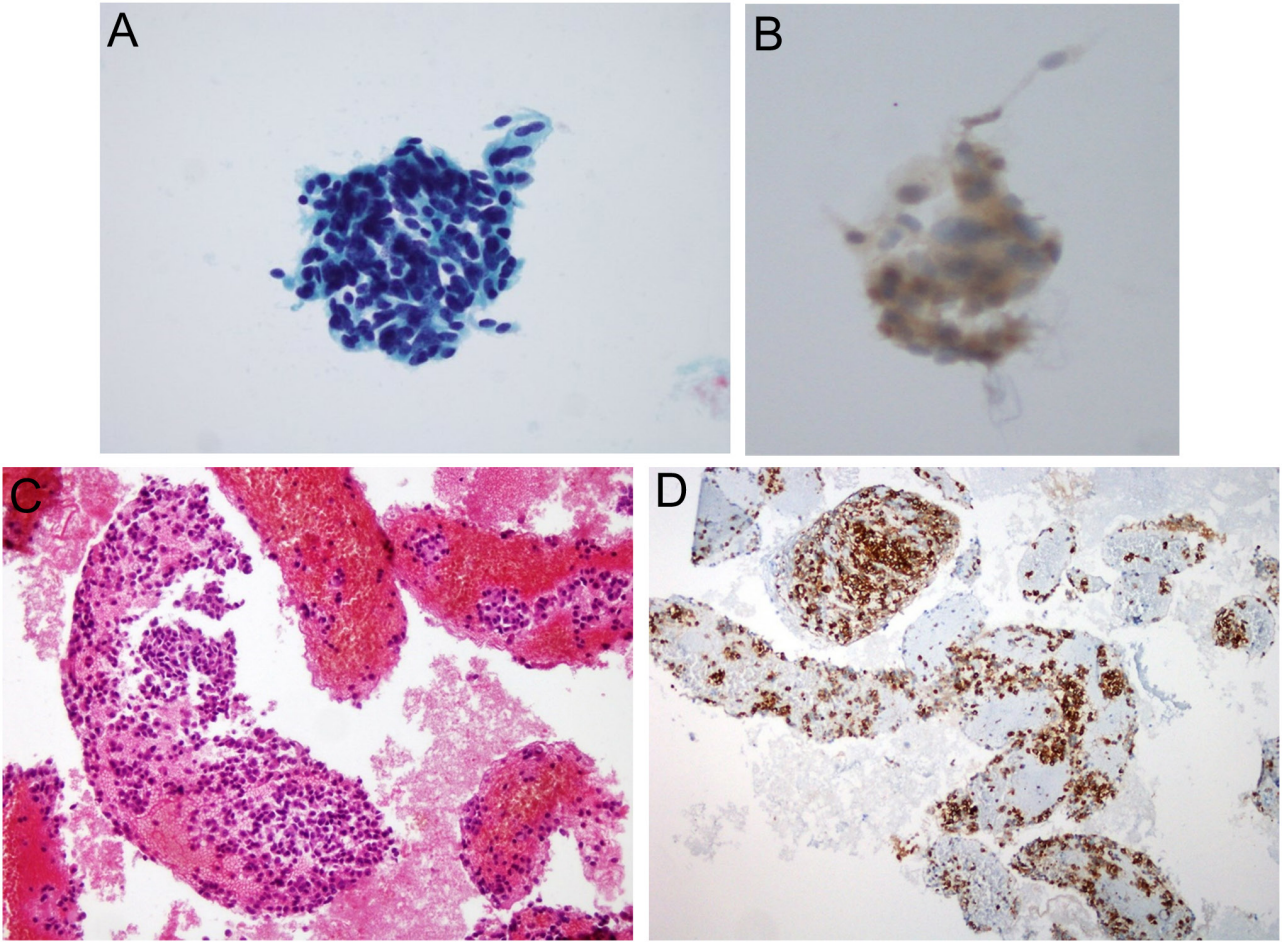
(A) Neuroendocrine tumor. In LBC material, tumor cell plaques with hyperchromatic, pyknotic nuclei, narrow cytoplasm, and some spindle appearance (Papanicolaou stain; 20× magnification), (B) Pale cytoplasmic synaptophysin positivity in the immunohistochemical staining performed for tumor type determination in the cell plates in the LBC material, (C) Tumor cells with eccentric nuclei, monotonous, without prominent nucleoli, on blood and fibrinoid background in the cell block material of the same case (hematoxylin–eosin stain; 20× magnification), (D) Synaptophysin positivity in tumor cells in the cell block material of the same case.

**Figure 4.**
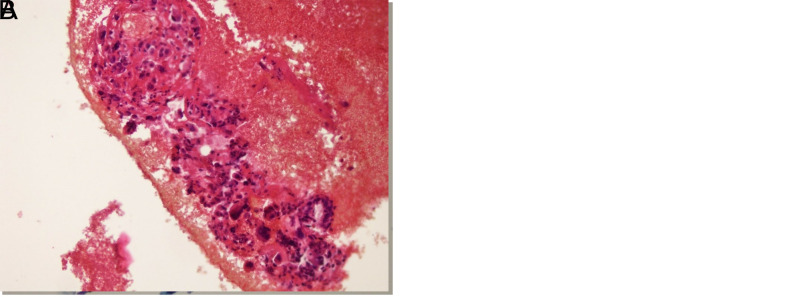
(A) Undifferentiated carcinoma. Pleomorphic, isolated tumor cells with bizarre nuclei in LBC material (Papanicolaou stain; 40× magnification), (B) In the cell block material of the same case, yellow pleomorphic, peculiar tumor cells, identical to the cells in the LBC material (hematoxylin–eosin stain; 40× magnification).

Although most patients (73% and 72.1% who underwent CB and LBC, respectively) were diagnosed with pancreatic adenocarcinoma, 17 were diagnosed with neuroendocrine tumor, 5 with lung adenocarcinoma metastasis, 3 with diffuse large B-cell lymphoma, 2 with solid pseudopapillary neoplasia, 1 with renal cell carcinoma metastasis, 1 with gastrointestinal stromal tumor, and 1 with malignant metastatic melanoma (Figures 2-4).

## Discussion

Despite the increasing use of FNB for EUS-guided tissue acquisition, FNA continues to be an important and valuable technique because of the disadvantages of FNB, including its lack of availability in many centers and high price. In this study, we evaluated the diagnostic efficacy of CB and LBC in EUS-FNA for diagnosing pancreatic tumors and found that the combined use of both methods resulted in higher diagnostic accuracy than the use of CB and LBC alone (*P* < .001). Notably, in cases where lesions in patients with suspicious for malignancy were considered malignant, a separate analysis was performed. This analysis also revealed that the combined use of both methods increased the diagnostic accuracy (*P* < .001) (Table 2).

Early detection of pancreatic cancer significantly affects its treatment and prognosis. The chances of surgical resection and survival rates decrease with advancements in tumor stage and size.^17^ The diagnostic sensitivity of cytologic and/or histologic specimens obtained from pancreatic lesions using EUS-FNA is affected by the experience of the endoscopist performing the procedure, the experience of the pathologist evaluating the specimen, the characteristics of the lesion (size/location), and the size and shape of the fine needle through which the specimen is taken. The procedure used for sample processing and the quality of the sample slides (cellular overlap, dryness, blood cells, and contamination) are the most important factors that affect the sensitivity.^18-20^ Diagnostic sensitivity, specificity, and accuracy of EUS-FNA in pancreatic solid tumors are 85%-98%, 95%-100%, and 91%-98%, respectively.^3,4^ In this study, the combined use of CB and LBC yielded a diagnostic accuracy of 98% when the results of the patients with suspicious for malignancy were excluded and 98.2% when the patients with suspicious for malignancy were considered malignant. Furthermore, these results were similar to those reported in the literature.

Smear cytology, a traditional and standard method for cytologic diagnosis, and performing ROSE significantly reduces the number of inconclusive samples, increasing diagnostic sensitivity and overall accuracy.^18,21,22^ However, in many developing countries, it is difficult to have an on-site cytopathologist for every patient due to financial constraints. Liquid-based cytology requires less skill than conventional SC preparation and offers favorable cellular preservation. Liquid-based cytology has been accepted as the preferred specimen collection method in the absence of ROSE because it is easy to use and has a diagnostic accuracy equivalent to that of SC prepared with ROSE,^22^ even when performed by endoscopists who are still learning.^13,23^ However, LBC is a thin-layer slide preparation method, and it is particularly advantageous for cytopathologic evaluation because it overcomes the disadvantages of SC including cell crowding and blood contamination, reveals more cellularity with a cleaner background and better cytomorphologic features, and can be used to conduct immunocytochemistry and molecular biology studies.^21,24-27^ In this study, the diagnostic accuracy of LBC was 87.9% and 89% when cases with suspicion for malignancy were excluded and considered malignant, respectively. Some studies reported diagnostic accuracy up to 88-90.2% by LBC.^28,29^ Our results are correlated with the published literature.

Pathologists prefer CB over cytologic specimens because the artifact rate is lower in CB than in SC and the appearance of CB is much closer to the actual tissue architecture.^30^ Moreover, CB is more suitable than SC and LBC for immunohistochemical applications and is considered the gold standard of histological staining.^31,32^ However, CB samples are often fragmented and small and are rarely sufficient to establish a diagnosis when used alone.^33^ Therefore, the use of additional methods contributes to increased diagnostic accuracy. In this study, the diagnostic accuracy of CB alone was 92.4% when cases with suspicion for malignancy were excluded and 93.1% when they were considered malignant. When combined with LBC, the diagnostic accuracy of the two methods reached 98%. In 15 (4.4%) cases where CB was non-diagnostic and/or misdiagnosed, the correct diagnosis was achieved using LBC. Qin et al^34^ evaluated the diagnostic accuracy of CB, SC, and LBC (91.7%, 75%, and 77.8%, respectively) and reported that CB was superior to other methods and neither the combination of CB and SC nor CB and LBC increased diagnostic accuracy. However, the results of this study demonstrated that the diagnostic accuracy increased with the combined use of both methods.

In this study, the diagnostic accuracy of LBC was significantly lower in solid-cystic lesions than in solid lesions (*P* = .031). This highlighted the diagnostic challenges arising from the presence of necrotic cells in the cystic degeneration areas of solid tumors; furthermore, a combination of both methods would be recommended in these cases. In this study, 6.7% and 6.9% of the samples collected for CB and LBC, respectively, had cellular deficiency, which was lower than the deficiency rates found in SC, CB, and LBC samples (12.5%, 33.3%, and 41.7%, respectively) reported by Yeon et al.^35^

Combined use of LBC with CB is also found to be useful in the diagnosis of many other diseases, such as oral diffuse large B cell lymphoma, endometrial lesions, papillary thyroid carcinoma, and evaluation of peritoneal fluid in gynecologic malignancies. Therefore, the usage of combined cytopathological techniques should be increased in pancreatic disorders.^36-39^

Limitations of the present study are its retrospective nature and single-center study.

In conclusion, LBC and CB are equally important for the diagnosis of pancreatic tumors in patients undergoing EUS-FNA. The diagnostic accuracy of the combined use of both methods was significantly higher than that of LBC and CB alone. This is particularly important in centers where ROSE cannot be performed. The use of LBC in combination with CB may be considered a favorable choice to increase the diagnostic accuracy and reduce loss of time.

## Figures and Tables

**Table 1.** Demographic, Clinical Data, and Cell Block/Liquid-Based Cytology Results

	Mean (SD)	Median (Minimum–Maximum)
Age	64.24 (11.46)	66 (22-87)
Size of the lesion (mm)	39.88 (13.89)	39 (7-120)
	n	%
Sex		
Female	170	43.6
Male	220	56.4
Appearance of the lesion		
Solid	359	92.1
Solid-cystic	31	7.9
Location in the pancreas		
Head	195	50
Body	87	22.3
Tail	72	18.5
Uncinate process	25	6.4
Neck	11	2.8
Cell block diagnosis		
Malignant		
Adenocarcinoma	286	73.3
NET	17	4.4
Anaplastic carcinoma	5	1.3
Lung adenocarcinoma metastasis	5	1.3
Undifferentiated carcinoma	5	1.3
DLBCL	3	0.8
SPPN	2	0.5
GIST	1	0.3
RCC metastasis	1	0.3
Suspicious for malignancy	39	10 v
Negative for malignancy	1	0.3
Non-diagnostic	26	6.7
Liquid-based cytology diagnosis		
Malignant		
Adenocarcinoma	281	72.1
NET	12	3.1
Anaplastic carcinoma	5	1.3
Undifferentiated carcinoma	5	1.3
Lung adenocarcinoma metastasis	4	1
DLBCL	3	0.8
SPPN	2	0.5
RCC metastasis	1	0.3
Malignant melanoma metastasis	1	0.3
Suspicious for malignancy	44	11.3
Negative for malignancy	5	1.3
Non-diagnostic	27	6.9

DLBCL, diffuse large B-cell lymphoma; GIST, gastrointestinal stromal tumor; NET, neuroendocrine tumor; RCC, renal cell carcinoma; SPPN, solid pseudopapillary neoplasia.

**Table 2.** Evaluation of Results When Cases with Suspected Malignancy Were Considered Malignant and When Cases with Suspected Malignancy Were Excluded

	Malignancy Diagnosis n (%)		*P*
	False	True	
When cases with suspicion for malignancy were considered malignant			
CB diagnostic accuracy (A)	27 (6.9)	363 (93.1)	*P* (A–B) = .045
LBC diagnostic accuracy (B)	43 (11)	347 (89)	*P* (A–C) = .001
Combined method accuracy (C)	7 (1.8)	383 (98.2)	*P* (B–C) < .001
When cases with suspicion for malignancy were excluded*			
CB diagnostic accuracy (A)	27 (7.6)	324 (92.4)	*P* (A–B) = .059
LBC diagnostic accuracy (B)	43(12.1)	313 (87.9)	*P* (A–C) = .001
Combined method accuracy (C)	7 (2)	339 (98)	*P* (B–C) < .001

Pearson’s chi-square test (Monte Carlo).

CB, cell block; LBC, liquid-based cytology.

*39 cases were evaluated as suspicious for malignancy by CB and 34 cases by LBC, and statistical analysis was made according to the cytopathologic results of 351 cases for CB and 356 cases for LBC, without suspicious cytopathology result.

**Table 3.** Evaluation of Diagnostic Accuracy of Both Methods According to Other Parameters

	Combined Method Accuracy^a^		*P*	Combined Method Accuracy^b^		*P*
	False	True		False	True	
	n (%)	n (%)		n (%)	n (%)	
Sex			.704f			.463f
Female	2 (1.2)	168 (98.8)		2 (1.3)	155 (98.7)	
Male	5 (2.3)	215 (97.7)		5 (2.6)	185 (97.4)	
Appearance of the lesion			.999f			.999f
Solid	7 (1.9)	352 (98.1)		7 (2.2)	316 (97.8)	
Solid-cystic	0 (0.0)	31 (100.0)		0 (0.0)	24 (100.0)	
Location in the pancreas			.218ff			.196ff
Uncinate process	1 (4.0)	24 (96.0)		1 (4.5)	21 (95.5)	
Head	6 (3.1)	189 (96.9)		6 (3.5)	164 (96.5)	
Neck	0 (0.0)	11 (100.0)		0 (0.0)	11 (100.0)	
Body	0 (0.0)	87 (100.0)		0 (0.0)	79 (100.0)	
Tail	0 (0.0)	72 (100.0)		0 (0.0)	65 (100.0)	
	**M** **edian (q1/q3)**	**M** **edian (q1/q3)**		**M** **edian (q1/q3)**	**M** **edian (q1/q3)**	
Age	50 (43/69)	66 (57/73)	.032^U^	50 (43/69)	66 (57/73.5)	.024^U^
Lesion size (mm)	45 (20/50)	39 (30/47)	.962^U^	45 (20/50)	40 (30/47)	.975^U^

q1: percentile 25; q3: percentile 75.

^a^When malignant suspects are correctly considered.

^b^When malignant suspects are excluded.

^ff^Fisher–Freeman–Halton (Monte Carlo).

^f^Fisher exact test (exact).

^U^Mann–Whitney *U*-test (Monte Carlo).

**Table 4.** Evaluation of the Diagnostic Accuracy of Cell Block and Liquid-Based Cytology with Respect to Other Parameters

	CB Diagnostic Accuracy		*P*	LBC Diagnostic Accuracy		*P*
	False	True		False	True	
	n (%)	n (%)		n (%)	n (%)	
Sex			.412^E^			.199^E^
Female	25 (14.7)	145 (85.3)		28 (16.5)	142 (83.5)	
Male	40 (18.2)	180 (81.8)		48 (21.8)	172 (78.2)	
Appearance of the lesion			.325^E^			.031^E^
Solid	58 (16.2)	301 (83.8)		65 (18.1)	294 (81.9)	
Solid-cystic	7 (22.6)	24 (77.4)		11 (35.5)	20 (64.5)	
Location in the pancreas			.634ff			.091ff
Uncinate process	4 (16.0)	21 (84.0)		7 (28.0)	18 (72.0)	
Head	38 (19.5)	157 (80.5)		46 (23.6)	149 (76.4)	
Neck	2 (18.2)	9 (81.8)		1 (9.1)	10 (90.9)	
Body	12 (13.8)	75 (86.2)		10 (11.5)	77 (88.5)	
Tail	9 (12.5)	63 (87.5)		12 (16.7)	60 (83.3)	
	**M** **edian (q1/q3)**	**M** **edian (q1/q3)**		**M** **edian (q1/q3)**	**M** **edian (q1/q3)**	
Median (q1/q3)	65 (56/70)	66 (57/74)	.101^U^	63.5 (53.5/70)	66 (58/74)	.009^U^
Size of the lesion (mm)	35 (28/45)	40 (30/48)	.049^U^	37 (30/50)	40 (30/46)	.634^U^

CB, cell block; LBC, liquid-based cytology.

^E^Pearson’s chi-square test (Monte Carlo).

^U^Mann–Whitney *U-*test (Monte Carlo).

^ff^Fisher–Freeman–Halton (Monte Carlo).
